# Occupational stress and health risk of employees working in the garments sector of Bangladesh: An empirical study

**DOI:** 10.3389/fpubh.2022.938248

**Published:** 2022-08-16

**Authors:** Deli Yuan, Md. Abu Issa Gazi, Md. Alinoor Rahman, Bablu Kumar Dhar, Md. Atikur Rahaman

**Affiliations:** ^1^School of Management, Jiujiang University, Jiujiang, China; ^2^Department of Management, Islamic University, Kushtia, Bangladesh; ^3^Mahidol University International College, Mahidol University, Nakhon Pathom, Thailand

**Keywords:** stress, health risk, garments, occupation, employees

## Abstract

The present study was conducted with a view to examining the impact of occupational stress on employees' health risk. A total number of 350 garment employees (114 supervisors and 236 workers) were selected from 25 readymade garment factories of Dhaka, Narayanganj, and Gazipur industrial areas of Bangladesh on a random sampling basis. Occupational stress was estimated using an ERIs modified questionnaire; when self-reported health problems, work related information and socio-demographic information were obtained using face-to-face interviews using a pre-formed questionnaire. The survey was conducted for 2 years from January 2020 to December 2021 in Dhaka, Narayanganj and Gazipur districts where most of the garment industries in Bangladesh are located. All data were processed by using Statistical Package for Social Sciences (SPSS) and Decision Analyst Stats, Version 2.0. For analyzing data, suitable statistical tools such as two-way ANOVA, *z*-test, chi-square test, Pearson's product-moment correlation, stepwise multiple regressions, and descriptive statistics were used. The results of the present study reveal that the occupational stress had a significant positive influence on health risk. The findings also reveal that both the male and female employees perceived garment job highly stressful and risky for their health causes many dies and sickness, but it was higher among the female employees than their counterparts. Study suggests that due to major illness and diseases garments' employees are lacks of sound health that have to consider remedying for reducing occupational stress and health risk.

## Introduction

Bangladesh's ready-made garments industry started in the sixties. But in the last decade of the 1970s, this industry continued to grow as an export-oriented sector. Garment industry has been playing an impeccable role in the economy of Bangladesh as the number one sector in earning foreign currency (1) with the highest employment rate. Currently the garment industry accounts for 81.16 percent to total exports valued at USD 31456.73 million in the world as a largest export oriented industry of Bangladesh (2). Bangladesh's ready-made garment industry's strategy is to provide quality clothing at a low price to the world market. The demand for Bangladeshi garments in the international market is increasing day by day due to its producing quality garments and supplying them to the world at comparatively low prices. One of the reasons for the global attention of Bangladesh's garment industry is the empowerment of women and created employment opportunities for a large number of women workers (3). Over the past few decades Bangladesh's garment industry has been able to come to this position as a result of continuous development. The journey of the garment industry was no easy. The journey started with a handful of garment factories. In 1983, the number of garment factories was only 50, in 2000 it increased to 4,000 and in 2009 the number was 4,500 (3). And in the twentieth century the number has risen in FY 2019-20 of 500,000. The garment industry is also a major source of employment for a large number of people in this country. The garment industry in particular continues to play a huge role in employment of women. Eighty (80%) percent of workforce employed in the garment industry is women. The contribution of garment industry to the economy and GDP of Bangladesh is much higher than other sectors. The artisans behind it are a huge workforce in the garment industry, so garment workers are the pride of the nation and its women workers are recognized as the golden girl of the country. Four out of five of the 4.4 million workers employed in the garment industry in Bangladesh are women, so one can often consider issues facing this industry to be feminist issues. That is why the issues of safety, occupational stress and health risks of the labor force engaged in industry come up again and again especially after collapse Rana Plaza Building (4). We know that work and health are closely related. Most of the health problems faced by the garment workers are due to occupational stress including low wages, long working hours, reduced leave, and unhealthy working environment, working conditions, working in a crowded premise, misbehaver of supervisor and lack of safe health facilities. Stephen et al. (5) stated that the deleterious belongings of job stress are acknowledged as a challenge for both employers and employees. Work-related stress has been linked to numerous health hazards. The effects of work-related stress include the impact on workers' satisfaction, productivity, absenteeism, turnover and health (6). Occupational pressures are becoming increasingly important due to the constant changes the organizational structure (7). Garment workers feel a unique feebleness vulnerabilities in the workplace worldwide (8). However, health problems are the most significant among the vulnerabilities that workers face while working in the garment industry in Bangladesh (9). It is evident that Garments sector, workers of Bangladesh are the most affected by the unhygienic and unsafe nature of their workplace conditions (9–11). Nag et al. (12) revealed that the growing world market competitions are utterly affected the health of employees of garments sector that's in the context of Bangladesh. While physical work-related menaces like respiratory disease allied with occupational exposure (13) that have been studied in a limited number of prior researches in the field of RMG (14, 15), psychologically adverse work situations and their potential health effects have been inadequately addressed and have affected mental well-being (7, 16). Present study therefore set out with two objectives. Firstly, we aimed to analyze the level of occupational stress of the garments workers and secondly, to examine the impact of occupational stress on employees' health risk of garment industry in Bangladesh. In addition, in this research the interdisciplinary methodology used may be of concern to impend studies endeavoring to slant work stress as a racially entrenched phenomenon (17).

## Literature review

### Occupational stress

ILO define that occupational stress is the harmful emotional and psychological responses. Occupational stress is generally acknowledged by organizations that have trends of inefficiency, high turnover, due to absence illness, poor quality of work and increase healthcare costs and reduce employment satisfaction. Occupational stress is an emotional and physical condition, which affects person productivity, efficiency, personal health (18) and its quality work life. The experience of victims of occupational stress at work decreases performance and increase health risks (19, 20). Cao et al. (21) has described that Stress is described as, “a physical or psychological stimulus that can produce mental tension or physiological reactions that may lead to illness the occupational stress adversely affects the health and performance of the employees of an organization. Occupational stress is a complex psychological state of mystery. Occupational stress is a universal and common challenge to organization and employee productivity, it is the reality of modern day workplace (22), Stress contributes to decreased organizational performance, decreased employee overall performance, high error rate and poor quality of work, high staff turnover, and absenteeism (23). Based on Health and Safety Executive (24), role ambiguity, organizational change, job demands, bullying and violence are some of the common stress factors happening in the workplace today (25). Occupational stress is common among garments workers in Bangladesh due to many antecedents of stress (26). Redfearn et al. (27) revealed that occupational stress has a negative impact on life satisfaction (28); the study argued that occupational stress is an important factor in determining the life satisfaction and burnout levels (29). Occupational stress may have a negative effect on companies, such as increased absenteeism and employee turnover, decreased productivity and rising health care costs (30, 31). World Health Organization's (WHO) definition, occupational or work-related stress is the response people may have when presented with work demands and pressures that are not matched to their knowledge and abilities and which challenge their ability to cope. Denning et al. (15) defines stress as a physical, chemical, or emotional factor that causes physical or emotional arousal and can cause illness. This is a normal reaction when the brain receives a threat. When a threat is felt, the human body secretes hormones that activate its “fight or flight” response. Occupational stress can lead to a person's physical or mental condition in response to the workplace which creates a challenge for that employee. Causes of occupational stress include the environment, organizational climate, and the emergence of conflicts over employee job demands (32).

#### Demographic factor and occupational stress

Occupational stress is common among garments workers in Bangladesh due to many antecedents of stress (26). Occupational stress and demographic factors are related. Occupational stress of workers of garments industry is sometimes influenced by demographic factors. The availability of the studies among garments employees to detect issues that concomitant with work place stress remain a noteworthy challenges, outcomes from different studies showed that age (33, 34), education (35–37), experience (38, 39), marital status (37) and gender (34) significantly associated with occupational stress. Gebisa (33) found that age positively affect the occupational stress, high aged workers are felt high stress in the work place, it does not help denote positive influences as well as (35). Ajayi (40) argued that occupational stress has a positive association with gender; their study revealed that women workers suffer more from stress at work (41). In one survey 60% of employed women cited stress as their number one problem at work. Another study proved that in garment industry occupational stress are strongly linked to gender (42), and different hierarchical level of workers like workers suffer from greater work pressure than supervisors. Various studies have shown that occupational stress higher where the work experience is more, Personality factors have shown inclination toward stress (43). They also revealed stress related to the worker status and showed an association with education level and the occurrence of occupational stress. Another study (44) found that subordinate's workloads were associated with their leader's stress. Dey et al. (45) found that female workers had significantly more job stress than male workers and lowest salary ranges workers had significantly more job stress than highest salary ranges workers. Aderibigbe et al. (46) studied on occupational stress and found that graduate employees with more work experience expressed a significant higher level of occupational stress than their counterparts with less work experience [*t* = 4.43, df_(1, 530)_ *p* < 0.05]. Chandra and Parvez (47) studied effect of occupational stress and found that a negative relationship between demographic factors (age, experience, education and marital status) and stress.

Thus, we propose the following hypothesis:

*H1a. Demographic factors and occupational stress have a significant relationship between each other*.

*H1b. There is no relationship between level of employees and occupational stress*.

*H1c. There is no relationship between gender and occupational stress*.

#### Job related factor and occupational stress

There is a close relationship between occupational stress and job related factors. Various studies have demonstrated that a positive significant relationship is observed between occupational stress and job related factors like Pay (34, 38), Promotion (48), job status (49), Job security (37), working condition (38). Morke et al. (38) revealed that occupational stress negatively affects work safety, social security, interpersonal relationships in the workplace, conflict of responsibility and uncertainty, lack of autonomy and participation in the workplace, organizational arrangements and environment, career prospects, work and family balance problems, unequal work stress, health and safety risks, low wages etc. Most workers in the workplace face stress with work conditions work stress (50), little control over work; Role ambiguity and conflict, job insecurity; Bad relationships with colleagues and supervisors (51). Murali et al. (52) found that time pressure and role ambiguity have significant and negative influence on employee stress. Some of the recent findings unveiled that workload, time pressure, role conflict, lack of motivation, role ambiguity, reduction of resources, harassment, and many other factors impact employee performance (3). Time pressure seemed to become increasingly a main issue of work in most developing countries (22). Chaturvedi and Kumar (53) and Khan et al. (54) found that workload as a cause of occupational stress. As per Wang et al. (55) occupational stress is high due to the income level of the female workers in the garment sector is very poor. Ashton (56) found that workload, role conflict, and inadequate monitory reward are the prime reasons of causing stress in employees that leads to reduced employee efficiency. Predominantly, the garments sector was one in which workers were heavily influenced by work stress due to job uncertainty, long working hours, overtime, lack of administrative support. Therefore, employers need to identify the symptoms of work stress, and have the necessary knowledge and skills to manage and reduce the stress levels of their employees before the company itself is endangered (34). The study postulated that:

*H2. There is no relationship between job related factors and occupational stress*.

### Demographic factors, job factors and health risk

Workers faced numerous risks such as safety risks, mechanical risks, biological risks, ergonomic, physical risks and psychological risks (7). Many problems of garment workers are getting worse day by day due to occupational stress (23). That's why work environment should be stress free and safe, risks setting up and keeping up an unharmed workplace (57). Furthermore, according to Khan et al. (54) occupational safety and health can also reduce employee injury and illness related costs, including medical care, sick leave and disability benefit costs, etc. Talapatra and Rahman (23) and Chegini et al. (58) revealed some common health hazards of the garments factory premises including excessive sound and temperature, lighting and unclear working environment, exposure to undue vibration and dust, poor ventilation and work safety, and lack of disposal of wastes and effluents. Thatshayini and Rajini (59) also argued same types of health hazards in the garment industry which are liable to physical diseases and mental disorders. Legesse (60) noticed that the personality factors have shown inclination toward stress; stress causes anxiety, and other occupational health risk. It was found that working in garment factories harshly pretentious the health of workers because they were confined to a closed environment (53). The special nature of working on ready-made garments in the study area creates a variety of health risks. Many working conditions contribute to stress among women (61). Enyonam et al. (62) studied on health hazards of female garments' workers and noted that women workers in the garment sector mainly sew clothes and therefore have to breathe in the dust of the clothes which is a risk to their health. Dey et al. (45) showed that job stress was negatively correlated with mental health. Further, many of these health vulnerabilities arise from the nature of the RMG workplace, and include unhygienic and unsafe working environments, hazardous conditions of the factories, and lack of safety equipment (63). Chandra and Parvez (47) argued that workplace stress and female workers' health risk positively associated. Therefore, we proposed that:

*H3a. Demographic factors and health risk have a significant relationship between each other*.

*H3b. Job related factors and health risk have a significant relationship between each other*.

*H4a. Gender and health risk have a significant relationship between each other*

*H4b. Level of employee and health risk has a significant relationship between each other*

### Mediating effect of stress on health risk

Stress is a predictable cause for occupational injuries and health risks. Occupational stress is presently one of the most costly occupational health problems (64). Ornek and Sevim (65) suggested that occupational stress occurs when job demands and responsibilities are not commensurate with employees' abilities or when the time allotted for work is insufficient. Therefore, they cause many negative organizational consequences and unhealthy behaviors. Considered a public health problem due to its impact on workers' health, work-related stress can be seen as an illness that has emerged in modern society. Different reports indicate that workplace stress affects workers' health risk i.e., physical health, mental health, and behavior (33, 66, 67). Stress contributes to decreased organizational performance, decreased employee overall performance, high error rate and poor quality of work, high staff turnover, absenteeism and health risk. Due to occupational stress arise health problems such as anxiety, emotional disorder; work life imbalance; depression and other forms of ailments such as frequent headache; obesity and cardiac arrests. Study found that there are many cardiovascular disease related to stress, with being the main stress-related disease (68). Furthermore, levels of stress-related illness are nearly twice as high for women as for men. It has been observed that garment factory workers are confined in a closed environment which has a detrimental effect on their health. The special nature of the work done in the research area created a variety of health risks for selected respondents such as headaches, malnutrition, muscle aches, eye strain, loss of appetite, chest pain, back pain, unconsciousness, diarrhea, hepatitis (jaundice), food poisoning, asthma, fungal infections, helminthiasis, dermatitis and lose their eyesight (69). The Bureau of Labor Statistics in the US reported that in 2011, 58,860 job-place injuries and illnesses that made workers to absent from work occurred in Hospitals due to occupational stress. Sharif et al. (70) point out that female workers in the garment sector are more likely to feel overwhelmed by work stress, which poses a risk to health and ultimately leads to diseases such as asthma, shortness of breath, shortness of breath and conjunctivitis and visual discomfort. Occupational stress has increased risks of work-related diseases and accidents (71). Hafeez (72) argued that stress effects health and may lead to disease, Stressful working conditions can lead to behavioral, physical, and psychological strains. Stress related disorders encompass a broad array of conditions, including psychological disorders and physical diseases (blood pressure, headaches, diabetes, chronic pain, cardiovascular disease, and gastrointestinal disease. The present study also assessed mediating effect of occupational stress health risks. Thus, this study postulated that:

*H5. Occupational stress mediates the effect of health risk on (a) physical diseases (b) mental diseases*

*H6. Occupational stress and health risk have a significant relationship between each other*

**Figure 1:**
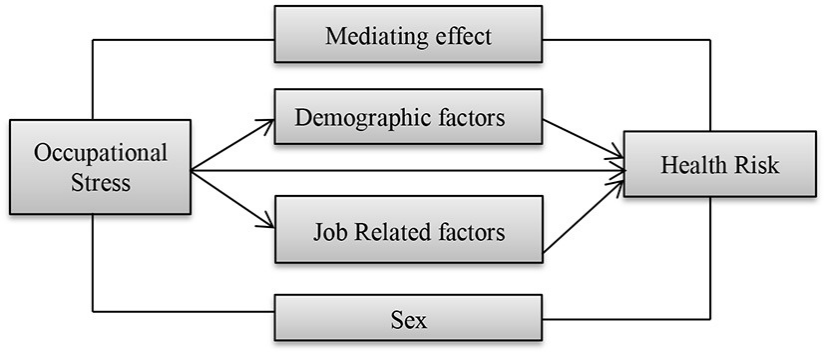
Research framework.

Based on the above literature review, this study attempts to draw the following proposed research framework. The proposed structure indicates the effect of occupational stress which ultimately affects the health risks of garment industry workers as shown in Figure 1.

This systematic review emphasis on understanding the effects of occupational stress and the health risks of garment workers. Therefore, this study was conducted to find out the occupational stress and health risks of workers in selected garment factories in Bangladesh due to the importance of occupational health.

## Research methodology

### Operationalization of constructs

The present study focuses whether occupational stress puts workers at heath risk. The study used Statistical Package for Social Sciences (SPSS) developed by Nie et al. (73). A structured questionnaire was used to collect the relevant data from the respondents which were; Scale for measuring occupational stress (SMOS) (36). Questionnaire for measuring health risk; and Open ended questionnaire for measuring major causes of diseases and major problems related to work. To measure the perceived job stress of the respondents, a single itemed 5-point scale consisting of simple statement (“Is your job stressful?”) was used. The respondents would indicate their feeling of stress by checking any one of the five categories of proposed pre-coded answers ranging from “not at all stressful” (1) to “Heavy stressful” (5). Higher scores indicate higher stress and the vice-versa. Different statistical tools ANOVA, Correlation, Regression etc. were used to test the hypothesis. Another measurement scales were developed on the basis of previous studies which are assumes to contribute to the edifice of occupational stress. One of the broadly used stress measurement scale is ERI that modified for this study (74) and measure the workers' occupational stress, Bengali version was used (75, 76). We used modified an eleven-item version of the Effort Reward Imbalance (ERI) (77) (Table 1).

**Table 1:** Overview of the work stress items Modified ERI and SMOS.

**Work stress items**	**Full questions**	**Source reference**
Pay	Are you stressed with the salary/wages that you draw from your present job?	ERI
Promotion	Are you stressed with the promotional opportunity at your present job?	ERI
Job status	Are you stressed with the job status at your present job?	ERI
Job security	Are you stressed with the job security at your present job?	ERI
Working condition	Are you stressed with the working condition of your present job?	ERI
Behavior of boss	Are you stressed with the behavior of your present boss?	ERI
Open communication	Are you stressed with the opportunity for open communication with your present boss?	ERI
Autonomy in work	Are you stressed with the autonomy in work at your present job?	ERI
Recognition for good work	Are you stressed with the recognition that is given for good work at your present job?	ERI
Participation in decision making	Are you stressed with the opportunity of participation in decision making at your present job?	ERI
Relation with colleagues	Are you stressed with the relation with colleagues at your present job?	ERI
**Full questions**	**Items**	**Source reference**
Is your job stressful?	Not at all stressful	SMOS
Is your job stressful?	Somewhat stressful	SMOS
Is your job stressful?	Quite stressful	SMOS
Is your job stressful?	Very much stressful	SMOS
Is your job stressful?	Extremely stressful	SMOS

Occupational stress assessment was performed using scientifically verified tools: Karasek's Work Content Questionnaire (JCQ) and Siegrist's Scale. In a combination of the two models, the author attempts to provide a comprehensive overview of multiple factors that affect health in the work environment. The health system included items on self-rated health (SRH) and self-reported physical symptoms (23, 78, 79). The response was delivered using a 5-point liqueur-scale (very good, good, medium, bad, very bad) of health of workers in garment factories in Bangladesh (26, 54).

### Survey administration and sample

The Garment Factories are mostly concentrated within the city limits of Dhaka, Chittagong, Narayangangj, and Gajipur in Bangladesh. Presently three types of garment factories are running in Bangladesh such as Woven Garment, Knit Garment, and Sweater Garment. To select the sample factories for the present study, two lists of total Garment Factories were collected from the Bangladesh Garment Manufacturers and Exporters Association (2) and Bangladesh Knit Manufacturers and Exporters Association (80). From these lists 25 Garment Factories were selected randomly as sample covering 10 Woven Garments, 12 Knit Garments, and 3 Sweater Garments factories from Dhaka, Narayanganj, and Gazipur districts of Bangladesh. To make the sample representative total 350 employees including 114 supervisors and 236 workers were selected from above 25 factories considering three sections of each (Table 2).

**Table 2:** Sample distribution according to type of organizations and level of employees (*N* = 350).

**Type of organizations**	**Level of employees**				**Total**	
	**Supervisor**		**Worker**		**(*****N*** = **350)**	
	**No**.	**%**	**No**.	**%**	**No**.	**%**
Woven	51	14.6	109	31.1	160	45.7
Knit	55	15.7	105	30.0	160	45.7
Sweater	8	2.3	22	6.3	30	8.6
Total	114	32.6	236	67.4	350	100

Several important factors have been considered behind the select of the proposed research areas. For example, firstly, selected areas are known as the estate of garments industry. In 2019, there were about 4.62 thousand garment factories in Bangladesh 85 percent of them are in Dhaka, Narayanganj, and Gazipur, published by Bangladesh Bureau of Statistics Department, March 29, 2021 (81). Secondly, garment factories have been established in all these selected places historically and in the generation to generation inheritably. Thirdly, due to the densely populated areas and the predominance of poor class peoples, there are advantages in providing low to those whose socio-economic status is not better. Formula by Andrew Fisher written permission was taken from the concerned authority of each of the selected garment factories. After getting permission each respondent was contracted personally, and data were collected individually from the respondents after making him/her convinced about the objectives of the study. The sittings were arranged in a suitable room provided by the authority of the factory concerned. Data were collected during the period from July to December 2020 by the researchers. When the respondents faced any problem, necessary clarifications were given to them. Employees with less than 2 years of experience were excluded. The interviews were taken during the working hours, lunch hours, and after office hours also. Each subject took about 40–50 min to finish the necessary information required to fill-up the questionnaire.

### Reliability and validity

Cronbach's consistency coefficient was employed in this study to examine the reliability and Fornell and Larcker's (82) convergent and discriminant validity test was used to investigate validity of the present study.

According to Cronbach's Alpha results, if the Coefficient Alpha is greater than 0.6 and the Composite Reliability is greater than 0.7, then the Purpose of Scale and questionnaire Used will be reliable. According to Table 3, Occupational Stress and Health Risk Related Measurement Scale and questionnaire have been found fit. In the case of occupational stress, Cronbach Alpha and composite reliability are 0.849 and 0.626, respectively. On the other hand, Cronbach Alpha 0.698 and Composite Reliability 0.69 in case of Health Risk.

**Table 3:** Reliability indices.

**Components**	**Cronbach's alpha**	**Composite reliability**	**Description**
Occupational stress	0.749	0.827	Reliable
Health risk	0.698	0.769	Reliable

According to Fornell and Larcker's formula, the AVE must be at least 0.5 and above for convergent tests. Thus the AVE value of Table 4 accepts convergent validity. On the other hand, the discriminant validity test is also valid because the diagonal value is higher than the off diagonal value.

**Table 4:** Validity analysis.

**Components**	**AVE**	**1**	**2**
1. Occupational stress	0.658	0.794	
2. Health risk	0.611	0.762	0.831

### Ethical statement

Prior to the commencement of the present study, the approval of the researchers from the academic institution was optional, but they agreed verbally during discussions with the heads of the departments concerned. Permission was obtained from the Bangladesh Garment Industry Regulatory Authority BGMEA during the collection of research data. All participants are asked whether they are willing to participate independently and voluntarily when collecting information in person. They voluntarily provide information without any interference. Necessary information is collected without identifying the names of the participants to ensure that the information provided will be used for research purposes only. Non-interested people are also applauded. However, the research team that assisted the helpers in data collection respectfully acknowledged their time and labor and provided compensation in certain cases. However, the research team recognizes the time and labor of the helpers in respecting the data collection and provides compensation in certain cases.

## Data analysis and results

### Socio-demographic information of the respondents

It is revealed from Table 5 that 56.00 percent of the respondents were male and the rest 44.00 percent were female. Among the supervisors, 73.68% were male and the remaining 26.32% were female. On the other hand, among the workers 47.46% were male and rest of the 52.54 was female. Among the total respondents, 52.3% were unmarried and the remaining 47.7 percent were married. Among the supervisors, 55.26% were married and rest of the 44.74% was unmarried. On the other hand, among the workers 55.93% were unmarried and the remaining 44.07 % were married. Furthermore, Table 5 reveals that the highest percentage (44.86%) of the respondents was from 22 to 29 age groups. Among the supervisors was 49.1% from 22 to 29 age group. On the other hand, among the workers the highest percentage (44.86%) was from 14 to 21 age groups. It was also observed that average age of the supervisors was higher (27.15) than that of the workers' (23.14).

**Table 5:** Socio-demographic profile of the respondents.

**Characteristics**	**N**	**%**	**Characteristics**	**N**	**%**
**Gender**			**Marital status**		
Male	196	56.00	Married	167	47.7
Female	154	44.00	Unmarried	183	52.3
Total	350	100.0	Total	350	100.0
**Education**			**Age**		
Illiterate	30	8.57			
Class 1–5	125	35.71	14–21 years	127	36.28
Class 6–9	96	27.43	22–29 years	157	44.86
S.S.C.	53	15.14	30–38 years	56	16.00
H.S.C.	36	10.29	30 and above	10	2.86
Degree and above	10	2.86	Total	350	100
Total	350	100			
**Experiences**			**Monthly income**		
2–5 years	153	43.72	Tk.5000–Tk7500	248	70.86
6–9 years	125	35.71	Tk.7501–Tk10000	75	21.43
10–13 years	52	14.86	Tk.10001–Tk12500	15	4.28
14 and above	20	5.71	Tk.12501 and above	12	3.43
Total	350	100	Total	350	100

Again, Table 5 shows that 8.57% of respondents were fully illiterate among 12.3% of the workers was fully illiterate, and the highest number of the respondents (35.71%) was from Class 1–5 (class I-V) 1–5 years of schooling group. It appeared from the Table 5 that the highest (248) number of respondents, (70.86%) income level were Tk.5000–7500 of the supervisors' was higher (3392.54) than that of the workers (3236.02). It is also observed that the second dominating income group (82) of the supervisors and workers was from TK. 7501–10000 (21.43%). Lastly, It is seen from Table 5 that the highest number (153) of the respondents (43.72%) were from 2 to 5 years of experience group where supervisors (48.2%) and workers (41.5%).

### Statistical assessment and results

To see whether there is any significant difference and association between occupational stress and demographic factors Siegrist Scale was applied (Table 6).

**Table 6:** Occupational stress based on Siegrist Scale and distribution of demographic factors.

**Variables**	**No stress N (%)**	**Stress N (%)**	**Mean**	**S.D**.	**z**	**Chi-square**	***P*-value**
**Age**							
14–30	103(30.29)	237(69.71)	78.55	7.00	−2.26	29.92	<0.05
30 above	2(20)	8(80)	60.26	7.11			
**Experience**							
2–13 years	111(33.64)	219(66.36)	69.43	7.35	0.284	1.819	<0.01
14 and above	5(25)	15(75)	48.21	6.66			
**Level education**							
Literate to HSC	112(32.94)	228(67.06)	89.32	6.24	−0.078	119.52	<0.05
Graduation and above	3(30)	7(70)	59.38	8.00			
**Level of employee**							
Workers	50(21.19)	186(78.81)	60.49	7.53	2.12	-	<0.05
Supervisor	71(62.28)	43(37.72)	58.79	6.82			
**Marital status**							
Married	93(55.69)	74(44.31)	59.76	6.41	1.05	-	NS
Unmarried	42(22.95)	141(77.05)	58.96	7.66			
**Gender**							
Male	82(41.84)	114(58.16)	3.13	1.56	−6.83	-	<0.01
Female	14(9.09)	140(90.91)	4.11	0.097			
**Income**	118(33.71)	232(66.29)	59.25	6.72	−0.359	-	<0.05

The results of the Table 6 suggest that there is a significantly associated with age (*P* ≤ 0.05) those who are comparatively young age (Mean = 78.55) the felt more occupational stress than higher age (mean = 60.26). According to Siegrist Scale the strongest association observed when considering the level of employees (*P* ≤ 0.01) and mean difference between job stress of the workers and supervisor are 60.49 and 58.79 as respondents. Occupational stress is additionally linked to level of education. The application of Siegrist Scale showed that significantly (*p* ≤ 0.05) more stress are related to the low educational level (mean = 89.32) but higher educated (mean = 59.38) also have occupational stress (*z* = −0.078). Furthermore, low income level employees are more stressed (66.29) than high income and statistically significant (*p* ≤ 0.05). The results also show that the mean difference between job stress of the male and female respondents. The direction of the results indicates that occupational stress was significantly (*p* ≤ 0.01) higher among the female respondents than that of their counterparts. But marital status and occupational stress are not significantly associated even though unmarried (77.05%) employees are more stressed than married. The Result of the Table 6 also reveals that there was a significant mean difference of occupational stress between the supervisors and workers. The direction of the results indicates that occupational stress was significantly higher among the workers than that of the supervisors.

The response patters of the respondents according to their occupational stress also assessed and the results are show in Table 7.

**Table 7:** Response patterns of the employees on the Occupational Stress based on Scale for measuring occupational stress (SMOS); (*N* = 350).

**Response patterns**	**Woven garment**		**Knit and sweater garment**		**Total respondents**	
	**(*****N*** **= 160)**		(***N*** = 190)		**(*****N*** **= 350)**	
	**Supervisor**	**Worker**	**Supervisor**	**Worker**	**Supervisor**	**Worker**
	**(*N* = 51)**	**(*N* = 109)**	**(*N* = 63)**	**(*N* = 127)**	**(*N* = 114)**	**(*N* = 236)**
Not at all	0	1	1	61	1	62
stressful	0	−0.9	−1.6	−48	−0.9	−26.3
Some what	1	1	0	18	1	19
stressful	−2	−0.9	0	−14.2	−0.9	−8.1
Quite	3	11	3	4	6	15
stressful	−5.9	−10.1	−4.8	−3.1	−5.3	−6.4
Very much	30	41	48	30	78	71
stressful	−58.8	−37.6	−76.2	−23.6	−68.4	−30.1
Extremely	17	55	11	14	28	69
stressful	−33.3	−50.5	−17.5	−11	−24.6	−29.2
Total	51	109	63	127	114	236
	−100	−100	−100	−100	−100	−100
**Response patterns**			**Total respondents (*****N*** **= 350)**		***Z*** **-value**	***p***
			**Yes**	**No**		
1. Not at all stressful			1(0.9)	62(26.3)	0.588	(P < 0.05)
2. Somewhat stressful			1(0.9)	19(8.1)	0.342	(P < 0.05)
3. Quite stressful			6(5.3)	15(6.4)	0.648	(P < 0.05)
4. Very much stressful			78(68.4)	71(30.1)	4.679	(P < 0.05)
5. Extremely stressful			28(24.6)	69(29.2)	0.397	(P < 0.05)

*Figures in the parentheses indicate percentage*.

The above Table 7 shows that 68.4% of the supervisors expressed that their job was very much stressful and 30 and 29% of the workers expressed that their job was very much stressful and extremely stressful, respectively. On the other hand, 58.8 and 76.2% of the supervisors of Woven & Knit and Sweater Garments expressed that their job was very much stressful, respectively. But, 50.5% of the Woven Garments workers opined that their job was extremely stressful while, 48% of the Knit and Sweater Garments workers perceived that their job was not at all stressful. Table 7 also shows that respondents opined regarding stress related questions, only 1 (0.9) respondent agreed that not at all stressful in garment industry. But all the workers have admitted that there is more or less work in the garment industry. However, a large number of workers (68.4%) opined that the occupational stress in the garment industry is too mush (very much stressful). The results suggested statistically significant of all items of response pattern for measuring occupational stress (*P* < 0.05).

The comparisons between the type of organizations (Woven and Knit and Sweater Garment) and level of employees (Supervisors and Workers) on job stress, and the ANOVA results to these effects are presented in Table 8.

**Table 8:** Summary of two-way ANOVA for nature of occupational stress by type of organizations and level of employees (*N* = 350).

**Sources of variation**	**Sum of square**	**df**.	**Mean square**	***F***	***P***
Main effects (combined)	229.26	2	114.63	98.20	<0.01
Type of organizations	168.41	1	168.45	144.27	<0.01
Level of employees	60.85	1	60.85	52.13	<0.01
2-way interactions	64.98	1	64.98	55.67	<0.01
Residual	403.88	346	1.17		
Total	698.12	349	2.00		

A close study of Table 8 reveals that the main effects and the interactions, if any, between type of organizations (Woven and Knit and Sweater) and level of employees (Supervisor and Worker) on job stress. It is evident from the results that F-ratios for two-way interactions and for the main effects of type of organizations and level of employees were statistically significant. This means that both the independent variables individually and interactionally produce significant difference on occupational stress.

Rates of agreement with the individual work stress items can be found in Table 9 shows that the rates of agreement with the individual work stress items. Pay, Job security and Participation in decision making were each stated by about 90% of the participants. It's observed that promotion, job status and autonomy in work support in either the foremen or workers were reported by about 70%. The results in the above table also indicate that significantly higher numbers of the respondents were stressed with their job security, pay, participation in management, promotional opportunity, autonomy in work, and job status. However, significantly higher numbers of respondents were not stressed with their behavior of boss, working condition, open communication, and recognition for work.

**Table 9:** Deals with individual occupational stress items (*n* = 350).

**Work stress items**	**Full questions**	**Source reference (Yes)**	**Source reference (No)**	***P***
Pay	Are you stressed with the	331	19	<0.01
	salary/wages that you draw from your present job?	−94.57	−5.43	
Promotion	Are you stressed with the	245	105	<0.01
	promotional opportunity at your present job?	−70	−30	
Job status	Are you stressed with the	217	133	<0.01
	job status at your present job?	−62	−38	
Job security	Are you stressed with the	338	12	<0.01
	job security at your present job?	−96.57	−3.43	
Working condition	Are you stressed with the	145	205	<0.01
	working condition of your present job?	−41.43	−58.57	
Behavior of boss	Are you stressed with the	139	211	<0.01
	behavior of your present boss?	−39.71	−60.29	
Open communication	Are you stressed with the	160	190	<0.01
	opportunity for open communication with your present boss?	−45.71	−54.29	
Autonomy in work	Are you stressed with the	233	117	<0.01
	autonomy in work at your present job?	−66.57	−33.43	
Recognition for good work	Are you stressed with the recognition that	166	184	<0.01
	is given for good work at your present job?	−47.43	−52.57	
Participation in decision making	Are you stressed with the	291	59	<0.01
	opportunity of participation in decision making at your present job?	−83.14	−16.86	
Relation with colleagues	Are you stressed with the	16	334	N. S.
	relation with colleagues at your present job?	−4.57	−95.43	

It is observed from the Table 9 that mean overall job satisfaction of the stressed respondents was significantly higher than that of those who were not stressed with the specific aspects of job, except only one job factor i.e., relation with colleagues.

To see whether there is any significant difference of the mean health risk of the level of workers (supervisors and workers) and, male and female respondents' *z*-test was applied and the results are shown in Table 10.

**Table 10:** Mean difference of health risk according personal factors of respondents based on Siegrist Scale (*N* = 350).

**Variables**	**No health risk N (%)**	**Health risk N (%)**	**Mean**	**S.D**.	***z***	**df**	***P*-value**
Respondents' opinion	34(9.71)	316(90.29)	175	199.40	11.80	348	<0.05
**Level of employee**							
Workers	16(6.78)	220(93.22)	118	144.25	10.08	358	<0.05
Supervisor	18(15.79)	96(84.21)	57	55.15	5.98		
**Marital status**
Male	19(9.69)	177(90.31)	98	111.72	8.378		<0.05
Female	15(9.74)	139(90.26)	77	87.68	7.94	358	

Table 10 showed that 90.29% of the respondents opined that the workers in the garment industry work under extreme health risks whereas only 9.71% of the workers believed that there is no such health risk that are statistically significant (*P* < 0.05). Furthermore, 220 workers (93.22%) orated they are at health risk and only 16 (6.78%) workers believed there is no health risk in the garment industry. On the other hand, total 96 (84.21%) supervisors commented that they work in the garment industry within health risk. However, the study found that the health risks of workers are higher than supervisors and was a significant difference between health risk of the workers and supervisors. The results of the Table 10 suggest that there was a significant mean difference between health risk of the male and female respondents. The direction of the results indicates that health risk was significantly higher among the female respondents than that of their counterparts.

The comparisons between the type of organizations (Woven and Knit and Sweater Garment) and basis of job stress on health risk, and the ANOVA results to this effect are presented in Table 11.

**Table 11:** Summary of two-way ANOVA for nature of health risk by type of organizations and basis of job stress of employees (*N* = 350).

**Sources of variation**	**Sum of square**	**df**.	**Mean square**	***f***	***P***
Main effects (combined)	558.502	2	279.251	694.050	<0.01
Type of organizations	168.405	1	168.405	418.553	<0.01
Basis of occupational stress	390.097	1	390.097	969.546	<0.01
2-way interactions	68.67	1	67.75	56.94	<0.01
Residual	139.615	347	0.402		
Total	698.117	349	2.000		

A close study of Table 11 reveals that the main effects (i.e., type of organizations and basis of occupational stress) were significant on health risk. The significant results suggest that type of organization and basis of occupational stress individually produce significant difference on health risk (*p* < 0.01).

The general illness as faced by the respondents according to the level of employees and type of organizations were seen and the findings are show in the Table 12.

**Table 12:** General illnesses as faced by the different category of respondents (*N* = 350).

**General illnesses**	**Woven garment (*****N*** = **160)**		**Knit and sweater garment** **(*****N*** = **190)**		**Total respondents (350)**	
	**Supervisor** **(*N* = 51)**	**Worker** **(*N* = 109)**	**Supervisor** **(*N* = 63)**	**Worker** **(*N* = 127)**	**Supervisor** **(*N* = 114)**	**Worker** **(*N* = 236)**
1. Headache	24	36	30	52	54	88
	(47.1)	(33.0)	(47.6)	(40.9)	(47.4)	(37.3)
2. Weakness	22	51	24	57	46	108
	(43.1)	(46.8)	(38.1)	(44.9)	(40.4)	(45.8)
3. Fever	8	26	14	32	22	58
	(15.7)	(23.9)	(22.2)	(25.2)	(19.3)	(24.6)
4. Cold and cough	7	27	10	22	17	49
	(13.7)	(24.8)	(15.9)	(17.3)	(14.9)	(20.8)
5. Eye trouble	17	20	23	34	40	54
	(33.3)	(18.3)	(36.5)	(26.8)	(35.1)	(22.9)
6. Body pain	21	43	21	34	42	77
	(41.2)	(39.4)	(33.3)	(26.8)	(36.8)	(32.6)
7. Stomach pain	3	14	3	13	6	27
	(5.9)	(12.8)	(4.8)	(10.2)	(5.3)	(11.4)
8. Mental pressure	38	87	29	93	67	180
	(74.51)	(79.82)	(46.03)	(73.23)	(58.77)	(76.27)

*i) Figures in the parentheses indicate percentage*.

*ii) Each respondent mentioned two major illnesses*.

It appears from the findings (Table 12) that the major illnesses as faced by the Woven Garment supervisors were: headache (47.1%), weakness (43.1%), body pain (41.2%) eye trouble (33.3%), and mental pressure (74.51). While, the major illnesses as faced by the Knit and Sweater Garment supervisors were: headache (47.6%), weakness (38.1%), eye trouble (36.5%), body pain (33.3%), and mental pressure (46.03). On the other hand, major illnesses as faced by the Woven Garment workers were: weakness (46.8%), body pain (39.4%), headache (33.0%), cold and cough (24.8%), and mental pressure (79.82). While, the major illnesses faced by the Knit and Sweater Garment workers were: weakness (44.9%), headache (40.9%), eye trouble, and body pain (26.8), and cold and cough (17.3%), mental pressure (73.23).

The diseases frequently attack the respondents according to the level of employees and type of organizations was also assessed and the results are show in the Table 13.

**Table 13:** Major diseases frequently attack the different category of respondents (*N* = 350).

**Major diseases frequently attack**	**Woven garment (*****N*** = **160)**		**Knit and sweater garment** **(*****N*** = **190)**		**Total respondents (350)**	
	**Supervisor** **(*N* = 51)**	**Worker** **(*N* = 109)**	**Supervisor** **(*N* = 63)**	**Worker** **(*N* = 127)**	**Supervisor** **(*N* = 114)**	**Worker** **(*N* = 236)**
1. Female diseases	4	10	0	1	4	11
	(7.8)	(9.2)	(0)	(0.8)	(3.5)	(4.7)
2. Diarrhea	6	6	5	15	11	21
	(11.8)	(5.5)	(7.9)	(11.8)	(9.6)	(8.9)
3. Jaundice	2	8	1	5	3	13
	(3.9)	(7.3)	(1.6)	(3.9)	(2.6)	(5.5)
4. Typhoid	2	8	1	3	3	11
	(3.9)	(7.3)	(1.6)	(2.4)	(2.6)	(4.7)
5. Urinal infection	0	6	0	1	0	7
	(0)	(5.5)	(0)	(0.08)	(0)	(0)
6. Pox	5	12	3	12	8	24
	(9.8)	(11.0)	(4.8)	(9.4)	(7.0)	(10.2)
7. No diseases	40	77	53	91	93	168
	(75.4)	(70.6)	(84.1)	(71.7)	(81.6)	(71.2)

*i) Figures in the parentheses indicate percentage*.

*ii) Each respondent mentioned two major diseases*.

It appears from the findings (Table 13) that 75.4% Woven Garment supervisors did not suffer from any diseases. The major diseases as suffered by the Woven Garment supervisors were diarrhea (11.8%), pox (9.8%), and female diseases (7.8%). While, (84.1%) Knit and Sweater Garment supervisors did not suffer from any disease. The major diseases suffered by the Knit and Sweater Garments supervisors were diarrhea (7.9%), and pox (4.8%). On the other hand, 70.6% Woven Garment workers suffered from no disease. The major diseases suffered by the Woven Garment workers were pox (11.0%), female diseases (9.2%), and jaundice & typhoid (7.3%). While, (71.7%) Knit and Sweater Garments workers suffered from no diseases. The major diseases as suffered by the Knit and Sweater Garments workers were diarrhea (11.8%), and pox (9.4%).

The interrelationships of some of the major variables of all 350 respondents (age, education, total income, work experience, nature of occupational stress, and health risk) find the nature and extent of the correlation that exists between those variables.

The results of the Table 14 reveal that there are significant positive correlations between age and total income, age and work experience, age and job stress, age and health risk, total income and education, total income and work experience, health risk and work experience; but there were significant negative correlations between education and work experience, job stress and total income, job stress and work experience. It also reveals that there is significant positive correlation between, job stress and health risk.

**Table 14:** Correlation matrix showing inter-correlations among some selected major variables (age, education, total income, work experience, nature of occupational stress, and health risk) of all the category of respondents taken together (*N* = 350).

**Variables**	**Age**	**Education**	**Total income**	**Work experience**	**Occupational stress**	**Health risk**
Age	1.00					
Education	0.099	1.00				
Total income	0.322 ^**^	0.156 ^**^	1.00			
Work experience	0.540 ^**^	−0.231^**^	0.420^**^	1.00		
Occupational stress	0.534 ^**^	0.103	−0.535^**^	−0.241 ^**^	1.00	
Health risk	0.414^*^	−0.054	0.017^**^	0.558^**^	0.567^**^	1.00

* *Correlation is significant at the 0.05 level (2-tailed)*.

** *Correlation is significant at the 0.01 level (2-tailed)*.

The results in Table 15 show that There was positive significant correlation between occupational stress and health risk (0.567^***^). Positive significant correlation had been found among almost all job facets and occupational stress except relation with colleagues and promotion. There was significant positive correlation among health risk and promotion, health risk and pay, health risk and job status, health risk and promotion, health risk and working conditions, health risk and behavior of boss, health risk and autonomy in work, and health risk and participation in decision making.

**Table 15:** Correlation matrix among dependent (occupational stress and health risk) and independent (job related factors) variables (*N* = 350).

**Variables**	**1**	**2**	**3**	**4**	**5**	**6**	**7**	**8**	**9**	**10**	**11**	**12**	**13**
1.Occupational stress	1												
2. Health risk	0.567 ***	1											
3.Pay	0.572***	0.338***	1										
4.Promotion	−0.217**	0.129*	0.205**	1									
5.Job status	413**	0.146*	0.185**	0.405**	1								
6. Job security	0.758***	0.069***	0.13 *	0.190**	0.385**	1							
7. Working condition	0.299**	0.267**	0.140*	0.183**	0.220**	0.276**	1						
8. Behavior of boss	0.208**	0.224**	0.111	0.001	0.116*	0.169**	0.483**	1					
9. Open communicat-ion	0.187**	0.143	0.115*	0.002	0.060	0.007	0.164**	0.432**	1				
10.Autonomy in work	0.321**	0.126*	0.097	0.046	0.103	0.169**	0.140*	0.13*	0.196**	1			
11.Recogniti-on for good work	0.129*	0.099**	0.085	0.195**	0.160**	0.061	0.208**	0.18**	0.225**	0.147*	1		
12.Participat-ion in decision making	0.278**	0.295**	−0.091	0.063	0.043	0.039	0.251**	0.280**	0.183**	0.174**	0.361**	1	
13.Relation with colleagues	0.044	0.041	−156**	−0.007	0.010	−0.019	0.061	0.021	0.013	0.033	0.043	0.130*	1

The * symbol indicate the correlation is significant at the 0.05 level (2-tailed).

The ** and *** indicate the correlation is significant at the 0.01 level (2-tailed).

To observe as consider the contribution of independent variables (occupational stress) on a dependent variable: health risk; inter-correlations between the variables were studied. The results of inter-correlation and contribution of independent variables on dependent variables are shown in Table 16.

**Table 16:** Correlation and regression analysis to explore the influence of Occupational stress on health risk.

**Variables**	**Job stress**	**Health risk**
Occupational stress	1	0.567^**^
Health risk	0.567^**^	1

** *Correlation is significant at the 0.01 level (2-tailed)*.

The results in the Table 16 indicates the correlation between health risk & occupational stress, it show the following significant correlation for workers: There was positive significant correlation between occupational stress and health risk. Positive significant correlation had been found between occupational stress and health risk.

Since, correlation between occupational stress and health risk is statistically significant, so linear regression analysis can be performed. These are shown in Table 17.

**Table 17:** Regression analysis to explore the influence of occupational stress on health risk (Regression analysis).

**Variable in the equation**	**R square**	**F statistic**	***P*-value**	**Beta**	***P*-value for Beta**
Occupational stress	0.334	90.806	0.000 <0.01	0.567	0.000 <0.01

Since, *P*-value of F-statistic is significant, so overall model is significant. In addition, significant *P*-value of Beta indicates that occupational stress has significant positive influence on health risk (Table 17).

## Discussion

There are 6 hypothesis were formulated on the basis of the review of literatures and in the light of the objectives of the present study stated earlier. The findings of the present study will be discussed in accordance with each hypothesis.

The results suggest that the out of five personal factors that are age, education, experience, and gender and income status had significant influence on job stress (35). Tables 6, 14 shows that there was significant difference between age group and stress level of the respondents; the results also indicated that the total 69.71% of the respondents was faced highly stress. The results revealed from Table 6 that there is no significant influence of the marital status on job stress but unmarried employees (141 = 77.05%) are more stressed and unhappy. Income level and occupational stress are found positive significant relationship. The work stress of low income workers is high whereas high income workers suffer less from work stress (43). The Tables 6, 14 also found that the stress level was significantly different among level of education. The table further implies that stress level was significantly different among level of experience. Thus, the results confirmed the 1st hypothesis. Several studies in home and abroad also found a significant influence of personal factors on the overall job stress e.g., Gebisa et al. (33), Jin et al. (34), Mathangi (37), and Aderibigbe et al. (46) which has confirmed the results of the present study.

Aderibigbe et al. (46) and Jin et al. (34) also found that have the co-relation between employee's personal profile and their stress with the job. Aderibigbe et al. (46) concluded that demographic factors have a role in occupational stress. The results of Table 6 showed that the job stress level was significantly different between workers and supervisor (*z* = 2.12). Out of 236 workers 186 (78.81%) workers opined they have serious occupational stress and out of 114 supervisors 43 (37.72%) supervisors' had work stress. The results in Table 7 showed that based on Scale for measuring occupational stress only 62 workers (26.3%) from three sections of garments industry opined that “not at all stressful” whereas only 0.9% supervisors expressed same opinion like workers. Furthermore, the results (Table 8) provided statistically significant recommendations for all items in the response pattern for occupational stress measurement (*p* < 0.05). The F-ratio was statistically significant (*p* < 0.01) for the two-way interaction and the major effects on the level of employees (workers and supervisors). Thus, the results rejected the null hypothesis 1b. Several studies such as Pindek et al. (44), Czuba et al. (31), and Redfearn et al. (29) found similar findings of the present study. Few studies (20, 21) revealed that less powerful employees are more likely to suffer stress than powerful workers and found high occupational stress in junior level. Islam et al. (26); found that in a workplace particularly stress-inducing are high- and low-status workers.

Study found that there is a relationship between gender and occupational stress. The results showed that mean job stress of the female employees was significantly greater than that of the male employees (Table 6), which rejected the null hypothesis number-1c i.e., the results confirmed the alternative hypothesis. Several studies also indicated higher job stress among the female employees than that of the male employees (42, 83). For instance, Khalid (42) found a significantly higher job stress among the female employees than that of the male employees. A female garment worker has to work from 8 a. m. to at least 8 p. m. in their working place (45). In addition, they are to perform their family duties. As a result, they get very little time for taking rest, which might affect both physical and mental health resulting in higher stress.

It is observed from the Table 9 that mean overall job stress of the respondents was significantly higher with the specific aspects of job, except only one job factor such as relation with colleagues. The results suggest that almost all job related factors (pay, open communication, job security, promotion, job status, participation in decision, working conditions, behavior of boss, autonomy in work and recognition for good work) had a significant influence on the job stress except relation with colleagues. Thus, the results rejected the 2nd null hypothesis and confirmed alternative hypothesis there is relationship between jobs related factors and occupational stress. Several investigators i.e., Jin et al. (34) and Jeong et al. (51) found a less or more significant impact of specific job factors on the overall occupational stress of the respondents, which confirmed the findings of the present study.

Demographic factors and health risk have a significant relationship between each other. Table 12 showed that the co-relation matrix indicates clearly the significant association between demographic factors and health risk. Study found positive correlation between health risk and age (0.414^*^), income (0.017^**^), and work experience (0.558^**^). Several studies found same results (7, 23, 53, 59). Thus, hypothesis 3a is accepted. Similarly, the hypothesis 3b also accepted because study found that job related factors and health risk had positive significant correlation. Results of the Table 15 have been found significant positive correlations between almost all aspects of work and professional stress, except relationships and promotion with colleagues. Several studies also supported same results and findings i.e., Mahmud et al. (84), Thatshayini and Rajini (59), Mohibullah et al. (57), Chaturvedi and Kumar (53), and Polat and Kalayci (85).

Table 6 showed that the gender and health risk have a significant relationship between each other. Among the male workers only 19 (9.69%) workers argued that there is no health risk whereas 177 (90.31%) workers believed there is health risk in garments industry. Again, among female workers those 139 (90.26%) workers noticed about health risk availability in the garments factories premises. Hence, the results confirmed the 4a hypothesis. Several studies also found the similar results. For instance, Chandra and Parvez (47) found women workers are more sufferers regarding health risk in garments factories. Some others Studies (59, 63, 84) which confirmed the findings of the present study. There is statistically significant health risks (*P* < 0.05) between supervisors and workers (Table 11). In addition, 220 workers (93.22%) said they were at health risk and only 16 (6.78%) workers believed there was no health risk in the garment industry. On the other hand 96 (84.21%) supervisors commented that they work in the garment industry at health risk (Table 10). Thus, the results confirmed the hypothesis noumber-4b. The findings of the present study supported by the results of some garment related studies [e.g., (7, 53, 54, 86)].

Occupational stress mediates the effect of health risk on physical diseases and mental diseases. The general major illnesses as faced by the respondents due to occupational stress were also studied (Table 12). Again in case of mental pressure total 58.77 percent supervisors and 76.27 percent workers are faced problem. It indicated that the general major illnesses as faced by both the groups were almost same. Several studies [e.g., (64, 67, 68, 78)] also found similar results, which confirmed the findings of the present study. Stressful occupation causes some major diseases, which frequently attacked the respondents, were also investigated. The respondents were asked to mention the two major diseases as faced by them. It appears from the findings that 81.6% supervisors and 71.2% workers were not attacked with any disease at all (Table 13). It indicated that the major diseases as faced by both the groups were almost same. In addition, female employees were suffering from female diseases. Several studies [e.g., (23, 65, 66, 72)] also found similar diseases as faced by the garment employees, which confirmed the findings of the present study. Thus the hypothesis no 5 is accepted (87–91).

Entire the study has proved that occupational stress and health risk have a significant relationship between each other. The results in Tables 11, 15, 16 showed that the direct positively significant association between occupational stress and health risk (0.567^**^). Correlation and regression analysis also supported same findings (Tables 16, 17). The findings of the present study supported by the results of some recent garment related studies [e.g., (18, 23, 26, 46, 49, 51, 71, 78)]. Hence, the results confirmed the 6th hypothesis.

## Conclusion

Garment industry is the single largest foreign exchange earner in Bangladesh. The garments made with the touch of modernity and tradition are not only earning foreign currency but also establishing Bangladesh in the world arena. It is undeniable that employment opportunities are being created for a large number of unemployed people especially women and their contribution to women's empowerment is undeniable. There are no good facilities including salary, there is no suitable healthy working environment. In addition, In addition, various work stressors exist everywhere. Lack of advantageous work environment, lack of proper healthy factory premises and incidence of mental abuse is one of the longest standing forms of the garment industry in Bangladesh. Different types of existing problems create work pressure in the garment industry. They suffer from constant work stress which is affecting their physical and mental health. Women workers have higher occupational stress and health risks than male workers and general workers suffer more health risks than supervisors. The present research is being conducted on garment workers to measure the workload of Bangladesh garment workers and to determine what kind of health risks they are facing as a result of work stress. Research has shown that personal factors and job factors are helpful in creating occupational stress. And stress is creating health risks all the time, that's why there are different types of physical diseases and mental problems. Study observed that the stress level was the lowest (30.29%) in the age group of 14–30 years respondents. The results further indicated that 80% of the respondents faced high stress and 20% in the respondents of age groups 30-above years. This study found everyone is worried about income (232 = 66.29%) and have significant relation with experience level. Most of the respondents were argued that they have moderate stress on regarding all education level. There was a significant mean difference in occupational strain between supervisors and workers. Results revealed that both of workers and supervisors are under occupational trauma. the reasons for higher job stress among the female employees were due to multiple roles played by the female employees in their lives. They had to work outside the home, and at the same time they were fully responsible for the household affairs. As to garment employees, these arguments are also applicable. Personal factors and health risk have a significant relationship. It is observed that female workers' health risk is higher than male workers. Results revealed that gender and health risk have a positive significant connection. The findings of the present study observed from Table 8 that only 9.71% respondents argued that there is no health risk but 316 (90.29) employees found health risk. Comparatively workers are victims regarding health risk than supervisors. The respondents were asked to mention two general major illnesses as faced by them. It appears from the findings that the major general illnesses as faced by the supervisors were: headache (47.4%), weakness (40.4%), body pain (36.8%), eye trouble (35.1%), fever (19.3%), and cold and cough (14.9%). On the other hand, major general illnesses as faced by the workers were: weakness (45.8%), headache (37.3%), body pain (32.6%), fever (24.6%), eye trouble (22.9%), and cold and cough (20.8%) The major diseases attacking by the supervisors were: diarrhea (9.6%), pox (7.0%), and female diseases (3.5%). On the other hand, the major diseases attacking the workers were: pox (10.2%), diarrhea (8.9%), jaundice (5.5%), and typhoid and female diseases (4.7%). Current research demonstrates that work stress affects health risks. Excessive stress plays a positive role in creating various health risks and mental problems. It is very unfortunate that the main driving force of the garment industry is the protection of the rights of the working class and no government or non-government organization has come forward to solve the problem. Immediate action is needed to reduce work stress and health risks by improving the work environment. The results of the present study will be able to give some idea about the occupational stress and health risks of the workers. We believe that by implementing these results, the policy makers, sociologists, psychologists, NGOs, women's rights organizations, social workers and the government will play a role in protecting the interests and health of the workers in this industry. This study was conducted at a time when there was an epidemic in the world which was not considered in this study.

## Limitations of the study and future research directions

Every scientific and social research has some limitations, considering the limitations the door to future extended research is opened. The current study is not out of bounds and paves the way for future research. There are also limitations to the questionnaire used in the present study. The factors that led to the collection and analysis of the data are largely old-fashioned. There are many more aspects of occupational stress that affect and accelerate stress. Researchers fail to consider possible unique stressors and dangerous working circumstances, which have a significant impact on occupational stress. It is suggested that particular stressors and concerns connected to occupational stress be assessed, including a comprehensive questionnaire covering various areas and dimensions of stress that may improve the interpretation and cross-referencing of data. Self-report based data collecting techniques were also used in this study to measure health risk. Self-report assessments have been shown to suggest biases like acquiescence. Several methodologically biased sources should be taken into account. Determining additional statistical techniques and taking into account the inclusion of adjuvant scales designed to evaluate and manage potential sources of bias that might affect the outcomes of prognostic and illustrative studies is what we advise for future field research and interventions. The survey was conducted on only 350 respondents in a large sector like garment industry which is one of the weaknesses of the study. Questionnaire and assessment scale was not modern. A longitudinal research might employ qualitative data from questionnaires or some more sophisticated approaches to gain a better understanding of what causes workplace stress. In the future, researchers will be able to provide better analytical results if they conduct research work considering a large number of samples, updated classical measurement scales, detailed facts and contemporary conditions. The present research work has not been financed sufficiently and has not been given enough time to complete. Due to limited funding and time allocation, Due to limited funding and time allocation, it was not possible to expand the research volume. As a result, the study was forced to complete a small number of samples in a short period of time. With adequate time and money allocated, the study would probably have had more scientific, promising accurate results.

## Data availability statement

The original contributions presented in the study are included in the article/supplementary material, further inquiries can be directed to the corresponding authors.

## Author contributions

Conceptualization: MG. Data curation: AR. Formal analysis and methodology: MG and AR. Investigation: DY and MAR. Project administration: DY and AR. Resources: BD and MAR. Validation and visualization: DY and BD. Writing—original draft: MG and AR. All authors contributed to the article and approved the submitted version.

## Funding

This research project was supported by the Foundation Project of National Natural Science Foundation of China (Grant Nos. 71962016 and 71962017).

## Conflict of interest

The authors declare that the research was conducted in the absence of any commercial or financial relationships that could be construed as a potential conflict of interest.

## Publisher's note

All claims expressed in this article are solely those of the authors and do not necessarily represent those of their affiliated organizations, or those of the publisher, the editors and the reviewers. Any product that may be evaluated in this article, or claim that may be made by its manufacturer, is not guaranteed or endorsed by the publisher.

## References

[1] World Trade organization. Occupational Health: Stress at the Workplace. (2021). Available online at: https://www.who.int/news-room/q-a-detail/ccupational-health-stress-at-the-workplace.

[2] BGMEA. Export Performance, Comparative Statement on Export of RMG and Total Export of Bangladesh. (2021). Available online at: https://bgmea.com.bd/page/Export_Performance (accessed on August 08, 2021).

[3] Ahmed S Khan MH Hossain I. Work stress and self-reported health problems in female ready made garment workers. Texila Int J Public Health. (2017) 5:1–12. 10.21522/TIJPH.2013.05.04.Art056

[4] Hosen I Al Mamun F Sikder MT Abbasi AZ Zou L Guo T. Prevalence and associated factors of problematic smartphone use during the COVID-19 pandemic: a bangladeshi study. Risk Manag Healthc Policy. (2021) 14:3797–805. 10.2147/RMHP.S325126 34548828 PMC8448157

[5] Stephen AF Rathnayake MA Degamboda S. A study on occupational stress and its implication on direct cadre employee turnover of Brandix Ltd. Int Res J Bus Manag. (2019) 7:1–10.

[6] Saleem F Malik MI Qureshi SS. Work stress hampering employee performance during COVID-19: Is safety culture needed? Front Psychol. (2021) 12:655839. 10.3389/fpsyg.2021.655839 34512434 PMC8426577

[7] Duran F Woodhams J Bishopp D. The relationships between psychological contract violation, occupational stress, and well-being in police officers. Int J Stress Manag. (2021) 28:141–6. 10.1037/str0000214

[8] Brown TA. Confirmatory Factor Analysis for Applied Research. 2nd ed. New York, NY: Guilford Publications (2015).

[9] Akram O. Occupational health, safety and extreme poverty: a qualitative perspective from Bangladesh. Int J Occupat Safe Health. (2014) 4:41–50. 10.3126/ijosh.v4i1.10654

[10] Chen YX Gao BA Cheng HY Li LF. Survey of occupational allergic contact dermatitis and patch test among clothing employees in Beijing. Biomed Res Int. (2017) 2017:3102358. 10.1155/2017/3102358 28396866 PMC5370485

[11] Chumchai P Silapasuwan P Wiwatwongkasem C Arphorn S Suwan-ampai P. Prevalence and risk factors of respiratory symptoms among home-based garment Workers in Bangkok, Thailand. Asia Pac J Public Health. (2015) 27:461–8. 10.1177/1010539514545647 25122551

[12] Nag A Murphy MJ Schulz W Cummins KL. Lightning locatingsystems: Insights on characteristics andvalidation techniques. Earth Space Sci. (2015) 2:65–93. 10.1002/2014EA000051

[13] Burgess MG Brough P Biggs A Hawkes AJ. Why interventions fail: a systematic review of occupational health psychology interventions. Int J Stress Manag. (2020) 27:195–207. 10.1037/str0000144

[14] Arslan G Yildirim M Tanhan A Bulus M Allen K-N. Coronavirus stress, optimism-pessimism, psychological inflexibility, and psychological health: psychometric properties of the coronavirus stress measure. Int J Ment Health Addict. (2021) 19:2423–39. 10.1007/s11469-020-00337-6 32837425 PMC7272108

[15] Denning M Goh ET Tan B Kanneganti A Almonte M Scott A. Determinants of burnout and other aspects of psychological well-being in healthcare workers during the Covid-19 pandemic: a multinational cross-sectional study. PLoS ONE. (2021) 16:e0238666. 10.1371/journal.pone.0238666 33861739 PMC8051812

[16] Schulz AD Schöllgen I Wendsche J Fay D Wegge J. The dynamics of social stressors and detachment: long-term mechanisms impacting well-being. Int J Stress Manag. (2021) 28:207–19. 10.1037/str0000216

[17] Sukla A Srivastava R. Development of short questionnaire to measure an extended set of role expectation conflict, coworker support and work-life balance: the new job stress scale. Cogent Bus Manag. (2016) 3:1134034. 10.1080/23311975.2015.1134034

[18] Rubin B Goldfarb R Satele D Graham L. Burnout and distress among allied health care professionals in a cardiovascular centre of a quaternary hospital network: a cross-sectional survey. CMAJ Open. (2021) 9:E29–E37. 10.9778/cmajo.20200059 33436453 PMC7843078

[19] Kar B Mishra B. A literature review on occupational stress and job performance. Int J Eng Manag Res. (2016) 6:402–7.19186902

[20] Patel AP„ Wang M Kartoun U„ Ng K Khera AV. Quantifying and understanding the higher risk of atherosclerotic cardiovascular disease among South Asian individuals. Circulation. (2021) 144:52430. 10.1161/CIRCULATIONAHA.120.052430 34247495 PMC8355171

[21] Cao W Hu L He Y Yang P Li X Cao S. Work-related musculoskeletal disorders among hospital midwives in chenzhou, hunan province, China and associations with job stress and working conditions. Risk Manag Healthc Policy. (2021) 14:3675–86. 10.2147/RMHP.S299113 34512055 PMC8423493

[22] Sumaryo P Ramly M Gani A Alam R. Effects of job stress, leadership on motivation and members of parliament of the regional house of representatives performance. Int J Humanit Soc Sci Invent. (2015) 4:49–54.

[23] Talapatra S Rahman MH. Safety awareness and worker's health hazards in the garments sector of bangladesh. Eur J Adv Eng Technol. (2016) 3:44–9.

[24] Health and Safety Executive. Work Related Stress, Anxiety, and Depression Statistics in Great Britain. (2016). Available online at: https://www.hse.gov.uk/statistics/ (accessed April 12, 2022).

[25] Zafar Q Ali A Hameed T Ilyas T Younas HI. The influence of job stress on employees performance in Pakistan. Am J Soc Sci Res. (2015) 1:221–5.35886597

[26] Islam MI Alam KMW Keramat SA. Working conditions and occupational stress among nurses in Bangladesh: a cross-sectional pilot study. J Public Health (Berl). (2021). 10.1007/s10389-020-01415-8

[27] Redfern JV Moore TJ Becker EA Calambodis J Hastings SP Irvine LM. Evaluating stakeholder-derived strategies to reduce the risk of ships striking whales. Divers Distrib. (2013) 25:1575–85. 10.1111/ddi.12958

[28] Ben-Zur H Michael K. Positivity and growth following stressful life events: Associations with psychosocial, health, economic resources. Int J Stress Manag. (2020) 27:126–34. 10.1037/str0000142

[29] Redfearn RA van Ittersum KW Stenmark CK. The impact of sensory processing sensitivity on stress and burnout in nurses. Int J Stress Manag. (2020) 27:370–9. 10.1037/str0000158

[30] Masoom MR Hoque MK. The effect of gender, age, experience and industry on employees' perceived stress: the case of Bangladesh. Romanian J Appl Psychol. (2018) 20:18–27. 10.24913/rjap.20.1.04

[31] Czuba KJ Kayes NM McPherson KM. Support workers' experiences of work stress in long-term care settings: a qualitative study. Int J Qual Stud Health Well Being. (2019) 14:1622356. 10.1080/17482631.2019.1622356 31156047 PMC6566720

[32] Mustafa UM Moni KN Rahman M. Social compliance, occupational health and environmental safety management practice in the apparel industry of Bangladesh: an overview. J Textile Sci Eng. (2018) 342, 2. 10.4172/2165-8064.1000342

[33] Gebisa G Wami SD Chercos DH Mekonnen TH. Work-related stress and associated factors among academic staffs at the university of gondar, northwest ethiopia: an institution based cross-sectional study. Ethiopian J Health Sci. (2020) 30:223–32. 10.4314/ejhs.v30i2.10 32165812 PMC7060383

[34] Jin W. Occupational stress and risk factors among workers from electronic manufacturing service companies in china. China CDC Weekly. (2020) 2:36. 10.46234/ccdcw2020.036 34594840 PMC8392886

[35] Gebisa G Sintayehu D. Perceived work-related stress and its associated factors among public secondary school teachers in Gondar city: a cross-sectional study from Ethiopia. BMC Res Notes. (2020) 13:36. 10.1186/s13104-020-4901-0 31952547 PMC6969455

[36] Selam G Balew Z. Workplace stress and associated factors among healthcare professionals working in public health care facilities in Bahir Dar City, Northwest Ethiopia, 2017. BMC Res Notes. (2019) 12:249. 10.1186/s13104-019-4277-1 31046816 PMC6498583

[37] Mathangi V. Impact of job stress on employees' job performance in Aavin, Coimbatore. J Organ Hum Behav. (2017) 6:21–9.

[38] Morke M Mulat G Destaw F. Work related stress and associated factors among Huajian shoe manufacturing employees in Dukem town, central Ethiopia. BMC Res Notes. (2018) 11:610. 10.1186/s13104-018-3727-5 30143059 PMC6109341

[39] Mansour S Mohanna D. Mediating role of job stress between work-family conflict, work-leisure conflict, and employees' perception of service quality in the hotel industry in France. J Human Resour Hospit Tourism. (2018) 17:154–74. 10.1080/15332845.2017.1340755

[40] Ajayi S. Effect of Stress on Employee Performance Job Satisfaction: A Case Study of Nigerian Banking Industry. (2018). Available online at: https://ssrn.com/abstract=3160620

[41] Almogbel Y. The effect of occupational stress on the quality of life of pharmacists in Saudi Arabia. Risk Manag Care Policy. (2021) 14:643–54. 10.2147/RMHP.S281317 33623454 PMC7896766

[42] Khalid A Pan F Li P Wang W Ghaffari AS. The impact of occupational stress on job burnout among bank employees in Pakistan. With psychological capital as a mediator. Front Public Health. (2020) 7:410. 10.3389/fpubh.2019.00410 32266193 PMC7105872

[43] Mulugeta H Tamene A Ashenafi T Thygerson SM Baxter ND. Workplace stress and associated factors among vehicle repair workers in Hawassa City, Southern Ethiopia. PLoS ONE. (2021) 16:e0249640. 10.1371/journal.pone.0249640 33819287 PMC8021151

[44] Pindek S Lucianetti L Kessler SR Spector PE. Employee to leader crossover of workload and physical strain. Int J Stress Manag. (2020) 27:326–34. 10.1037/str0000211

[45] Dey BK Rahman A Sultana MS Sadaf S. Garments worker's job stress and mental health. Int J Indian Psychol. (2016) 3:2348–5396. 10.25215/0304.013

[46] Aderibigbe JK Nwokolo EE Solomon O. Occupational stress among some Nigerian graduate employees: the impact of work experience and education. Cogent Psychol. (2020) 7:1802948. 10.1080/23311908.2020.1802948

[47] Chandra N Parvez R. Effect of stress on the health of women workers involved in garment manufacturing units. Int J Apparel Home Sci. (2019) 6:212–4.

[48] Panda K Philippe M. Occupational stress among textile workers in the democratic republic of congo. Trop Med Health. (2015) 43:223–31. 10.2149/tmh.2015-24 26865824 PMC4689605

[49] Sina TT Sintayehu KK. Occupational-Related musculoskeletal disorders and associated factors among beauty salon workers, adama town, south-eastern ethiopia. J Ergon. (2020) 9:257. 10.35248/2165-7556.20.9.257

[50] Useche SA Montoro L Alonso F Pastor JC. Psychosocial work factors, job stress and strain at the wheel: validation of the copenhagen psychosocial questionnaire (COPSOQ) in professional drivers. Front Psychol. (2019) 10:1531 10.3389/fpsyg.2019.01531 31312166 PMC6614297

[51] Jeong JG Kang SW Choi SB. Employees' weekend activities and psychological well-being via job stress: a moderated mediation role of recovery experience. Int J Environ Res Public Health. (2020) 17:1642. 10.3390/ijerph17051642 32138361 PMC7084709

[52] Murali SB Basit A Hassan Z. Impact of job stress on employee performance. Int J Account Bus Manag. (2017) 5:13–33.

[53] Chaturvedi LC Kumar A. A Study of Occupational Health Safety in the Garment Industry in Bangalore. (2015). Availabl eonline at: http://cividep.org/wp-content/uploads/2017/04/25-2-Occupational-healthsafety-13.pdf.

[54] Khan NR Dipti TR Ferdousi SK Hossain MZ Ferdousi S Sony SA. Occupational health hazards among workers of garment factories in Dhaka city, Bangladesh. J Dhaka Med Coll. (2016) 24:36–43. 10.3329/jdmc.v24i1.29560

[55] Wang Z Liu H Yu H Wu Y Chang S Lie Wang L. Associations between occupational stress, burnout and well-being among manufacturing workers: mediating roles of psychological capital and self-esteem. BMC Psychiatry. (2017) 17:364. 10.1186/s12888-017-1533-6 29141601 PMC5688661

[56] Ashton AS. How human resources management best practice influence employee satisfaction and job retention in the Thai hotel industry. J Human Resour Hospit Tourism. (2017) 12:1–25. 10.1080/15332845.2017.1340759

[57] Mohibullah AT Takebira UM Moni KN Rahman M. Social compliance, occupational health and environmental safety management practice in the apparel industry of Bangladesh: an overview. J Textile Sci Eng. (2018) 8:342.

[58] Chegini Z Jafarabadi MA Kakemam E. Occupational stress, quality of working life and turnover intention amongst nurses. Nurs Crit Care. (2019) 24:283–9. 10.1111/nicc.12419 30873678

[59] Thatshayini P Rajini PAD. Occupational safety and health hazards of apparel sector: perspective of northern province employees of Sri Lanka. J Bus Stud. (2018) 5:26–47. 10.4038/jbs.v5i1.23

[60] Legesse M. Impact of Occupational Safety Health on Organizational Performance in East Africa Bottling Sh. Co. East Africa. Addis: Addis Ababa University (2016). Available online at: http://etd.aau.edu.et/bitstream/123456789/12143/1/Muluken%20Legesse.pdf.

[61] Polat O Kalayci CB. Ergonomic risk assessment of workers in garment industry. In: Eight International Conference on Textile Science and Economy. Zrenjanin (2017). p. 16–21.

[62] Enyonam PA Gyamfi OA Emmanuel AK David B. The effect of occupational stress on job performance at aspet a. Company limited Global. J Arts Human Soc Sci. (2017) 5:1–17.

[63] Kabir H Maple M Usher K. Health vulnerabilities of readymade garment (RMG) workers: a systematic review. BMC Public Health. (2019) 19:70. 10.1186/s12889-019-6388-y 30646870 PMC6334416

[64] Hailemichael M Yefokir T Meaza G. Nonfatal occupational injuries among workers in microscale and small-scale woodworking enterprise in Addis Ababa, Ethiopia. J Environ Public Health. (2020) 2020:8. 10.1155/2020/6407236 32089714 PMC7013297

[65] Ornek OK Sevim E. Work-related stress and coping profiles among workers in outer garment sector; a cross-sectional study. Preprints. (2018) 2018:2018020061. 10.20944/preprints201802.0061.v1

[66] Zerihun A Abraham G Fekadu U. Occupational health risk of working in garages: comparative study on blood pressure and hematological parameters between garage workers and Haramaya University community,Harar, eastern Ethiopia. Risk Manag Healthc Policy. (2018) 2018:35–44. 10.2147/RMHP.S154611 29559815 PMC5856037

[67] Selam MN Ababu A Bayisa R Abdella M Diriba E Wale M. Prescribing pattern of dermatological compounding in Ethiopia: The case of alert hospital. Integr Pharm Res Pract. (2019) 11:1–8. 10.2147/IPRP.S346395 35024353 PMC8747791

[68] Serrano MÁ Costa R. Stressing the stress or the complexity of the human factor: psychobiological consequences of distress. In: García-Alcaraz J Alor-Hernández G Maldonado-Macías A Sánchez-Ramírez C, editors. New Perspectives on Applied Industrial Tools and Techniques. Management and Industrial Engineering. Cham: Springer (2018).

[69] Akhter S Rutherford S Chu C. Exploring the system capacity to meet occupational health and safety needs: the case of the ready-made garment industry in Bangladesh. BMC Health Serv Res. (2019) 19:435. 10.1186/s12913-019-4291-y 31253161 PMC6599266

[70] Sharif PA Islam ME Kabir RA. A study on occupational health and safety practices in RMG factories of bangladesh in accordance with compliance after rana plaza incident. Int J Bus Manag. (2015) 3:214.

[71] Mustafa M Illzam EM Muniandy RK Hashmi MH Sharifa AM Nang MK. Causes and prevention of occupational stress. IOSR J Dental Med Sci. (2015) 14:98–104.

[72] Hafeez S. The impact of job stress on performance of employees: a study of social security hospital of district okara and sahiwal. J Neuropsychol Stress Manag. (2018) 3:4–12.

[73] Nie NH Hull CH Jenkins JG Steinbrenner K Bent DH. Statistical Package for Social Sciences (SPSS). New York, NY: McGrow-Hill Book Company (1975).

[74] Siegrist J. Adverse health effects of high-effort/low-reward conditions. J Occup Health Psychol. (1996) 1:27–41. 10.1037/1076-8998.1.1.27 9547031

[75] Buapetch A Lagampan S Faucett J Kalampakorn S. The Thai version of effort-reward imbalance questionnaire (Thai ERIQ): a study of psychometric properties in garment workers. J Occup Health. (2008) 50:480–91. 10.1539/joh.L8017 18946191

[76] Li J Yang W Cheng Y Siegrist J Cho S-I. Effort-reward imbalance at work and job dissatisfaction in Chinese healthcare workers: a validation study. Int Arch Occup Environ Health. (2005) 78:198–204. 10.1007/s00420-004-0581-7 15838712

[77] Dragano N Siegrist J Wahrendorf M. Welfare regimes, labour policies and unhealthy psychosocial working conditions: a comparative study with 9917 older employees from 12 European countries. J Epidemiol Commun Health. (2011) 65:793–9. 10.1136/jech.2009.098541 20693497

[78] Aiggan T Hailemichael M Tesfaye A Steven MT. Musculoskeletal disorders and associated factors among vehicle repair workers in Hawassa City, Southern Ethiopia. J Environ Public Health. (2020) 2020:9472357. 10.1155/2020/9472357 32454844 PMC7229541

[79] Puja BP Henry H Kayvan M. Burnout assessment at a college of pharmacy, college of optometry, and school of physician assistant studies. Curr Pharmacy Teach Learn. (2021) 13:914–21. 10.1016/j.cptl.2021.06.010 34294254

[80] BKMEA. Bangladesh Knitwear Industry; Dynamics of Bangladesh Knitwear Sector. (2021). Available online at: https://new.bkmea.com/about-us/bangladesh-knitwear-industry/ (accessed on December 30, 2021).

[81] BBS. Report on the Six Case Studies on Selected Economic Activities 2020. Bangladesh Bureau of Statistics, Statistics and Information Division (2021). Available online at: http://bbs.portal.gov.bd/sites/default/files/files/bbs.portal.gov.bd/page/b343a8b4_956b_45ca_872f_4cf9b2f1a6e0/2022-05-11-08-37-f2bba97a8979397aa9ce8ddbbd59c588.pdf (accessed April 24, 2022).

[82] Fornell C Larcker DF. Evaluating structural equation models with unobservable variables and measurement errors. J Market Res. (1981) 18:39–50. 10.1177/002224378101800104

[83] Mostafa FH. Bangladesh: Toward Better Governance in the Ready-Made-Garment Sector, The Asia Foundation. (2021). Available online at: https://asiafoundation.org/2021/06/09/bangladesh-toward-better-governance-in-the-ready-made-garment-sector/ (accessed on August 08, 2021).

[84] Mahmud MS Mahmud R Jahan MN Hasan MR Rahman KM. Prevalence of health hazards: a study on the female workers of garment industry in Gazipur District, Bangladesh. J Appl Adv Res. (2017) 2:184–8. 10.21839/jaar.2017.v2i3.91

[85] Polat O Kalayci CB. Ergonomic Risk Assessment of Workers in Garment Industry. In: Eight International Conference on Textile Science Economy VIII. Zrenjanin (2016). p. 16–21.

[86] Tawiah KA Mensah J. Occupational health and safety and organizational commitment: evidence from ghanaian mining industry. Saf Health Work. (2016) 7:225–30. 10.1016/j.shaw.2016.01.002 27630792 PMC5011093

[87] Darvishpour M Hamidi M. The effect of mental stress on employees' job performance (case study of social security organization of khuzestan). Indian J Fundament Appl Life Sci. (2015) 5:2585–93.

[88] ILO. Work place Stress: A collective challenge. Geneva (2016). Available online at: https://www.ilo.org/safework/info/publications/WCMS_466547/lang–en/index.htm.

[89] Karasek R Brisson C Kawakami N Houtman I Bongers P Amick B. The job content questionnaire (JCQ): an instrument for internationally comparative assessments of psychosocial job characteristics. J Occup Health Psychol. (1998) 3:322–55. 10.1037/1076-8998.3.4.322 9805280

[90] Siegrist J Starke D Chandola T Godin I Marmot M Niedhammer I. The measurement of effort-reward imbalance at work: European comparisons. Soc. Sci. Med. (2004) 58:1483–99. 10.1016/S0277-9536(03)00351-4 14759692

[91] Sai Mei L Bhatti MA. Work stress and job performance in Malaysia academic sector: role of social support as moderator. Br J Econ Manag Trade. (2014) 4:1986–98. 10.9734/BJEMT/2014/12098

